# Glioma Stem Cells Upregulate CD39 Expression to Escape Immune Response through SOX2 Modulation

**DOI:** 10.3390/cancers14030783

**Published:** 2022-02-03

**Authors:** Bin Liu, Yufei Cao, Yanyan Li, Haifeng Ma, Mingfei Yang, Qiang Zhang, Guofeng Li, Kai Zhang, Yue Wu, Youxin Zhou, Wei Yang, Ting Sun

**Affiliations:** 1 Department of Neurosurgery, Laboratory of Brain and Nerve Research, The First Affiliated Hospital of Soochow University, Suzhou, Jiangsu 215006, China; liubin_1011@163.com (B.L.); yufei_cao@163.com (Y.C.); lyy0618@suda.edu.cn (Y.L.); neurosurgeryzk@163.com (K.Z.); wuyue_vic@163.com (Y.W.); brain_lab@suda.edu.cn (Y.Z.); 2 Department of Neurosurgery, Qinghai Provincial People’s Hospital, Xining, Qinghai 810007, China; mahaifeng_1013@163.com (H.M.); iloveyoucmu@163.com (M.Y.); zhangqiang691212@163.com (Q.Z.); liguofengxy@126.com (G.L.); 3 State Key Laboratory of Radiation Medicine and Protection, School of Radiation Medicine and Protection and Collaborative Innovation Center of Radiation Medicine of Jiangsu Higher Education Institutions, Soochow University, Suzhou, Jiangsu 215123, China

**Keywords:** CD39, SOX2, glioma stem cells, dendritic cells, extracellular ATP

## Abstract

**Simple Summary:**

Glioblastoma is the most malignant tumor of the central nervous system. Glioma stem cells are the cause of adverse outcomes such as early recurrence and low overall survival in glioma patients. Targeting glioma stem cells is considered a promising anti-glioma strategy, Although CD39 plays a key role in the initiation and regulation of DC-mediated antigen-specific immune responses, its impact on GSCs is unclear. Therefore, we systematically investigated the effect of CD39 on extracellular ATP levels, dendritic cell recruitment and T cell killing in glioma stem cells. The molecular mechanism by which SOX2 binds to the CD39 promoter to regulate extracellular ATP levels, and evaluated the immune response enhanced by inhibition of CD39 after ADM treatment in a mouse glioma model. We suggest that CD39 is an effective target for glioma immunotherapy.

**Abstract:**

Ectonucleotidase CD39 hydrolyzing extracellular ATP (eATP) functions as a key modulator of immune response in the tumor microenvironment, yet the role of CD39 in contributing tumor stem cells in a more immunosuppressive microenvironment remains elusive. Here we report that the upregulation of CD39 is crucial for the decrease of extracellular ATP concentration around glioma stem cells (GSCs) to maintain an immunosuppressive microenvironment. Adriamycin (ADM) is able to promote the release of ATP, which recruits dendritic cells (DCs) to phagocytose GSCs. CD39 inhibition further increased extracellular ATP concentrations following ADM treatment and DCs phagocytosis. In addition, GSCs upregulated CD39 expression by SOX2-binding CD39 promotor. In mouse tumor models, the combination of ADM and CD39 blockade increased immune cell infiltration and reduced tumor size. These findings suggest that GSCs upregulate CD39 expression by their biological characteristics to maintain an immunosuppressive microenvironment, and CD39 inhibition supplies a favorable tumor microenvironment (TME) for immunotherapeutic intervention and enhances the immune response induced by chemotherapy.

## 1. Introduction

Glioblastoma (GBM) is the most common primary malignant central nervous system tumor with a very poor prognosis [1]. Despite extensive surgical resection and adjuvant radiotherapy with temozolomide (TMZ) management, tumor recurrence always occurs. In recurrent GBM, the median overall survival is estimated to be 24–44 weeks [2,3]. Increasing evidence showed that the low survival rate is closely related to GSCs [4]. These infinitely self-renewing cells exist in a specific microenvironment. GSCs are resistant to conventional therapies and responsible for GBM relapse [5,6]. In the progress of cancer research, the discovery of immunotherapies provides new ideas for the treatment of glioma [7,8]. However, despite the significant therapeutic effect on several solid tumors, immune checkpoint blockade (ICB) has not shown any benefit in GBM treatment [9], and especially GSCs are insensitive to PD-1/PD-L1 treatment and characterized by immune escape [10]. Therefore, it is necessary to understand the molecular mechanism of GSCs in immune escape and find effective immunotherapy strategies.

CD39, also known as ectonucleotide triphosphate diphosphohydrolase-1 (ENTPD-1), is a rate-limiting enzyme that catalyzes ATP in a cascade reaction. ATP and ADP are converted to AMP, which is then converted to adenosine (Ado) by CD73 (ecto-5’-nucleotidase) [11]. Purinergic signals regulate inflammation and cancer, and play a key role in regulating cell migration, proliferation and death [12]. As the key molecules of purinergic signals, ATP and Ado in the TME are the most effective regulators of tumor cells and immune responses, and the ATP released by apoptotic cells is the main signal for the recruitment of immune cells [13]. In inflammation, hypoxia, ischemia and malignant tumors, and eATP levels increase significantly [14,15], however the synergistic enzymatic activity of CD39/CD73 pathway degrades eATP and produces immunosuppressive Ado in TME [16,17], which inhibits the anti-tumor immunity [18]. Blocking CD39 and other hydrolytic enzymes with an inhibitor such as POM-1 can enhance anti-tumor immunity and reduce metastatic spread [19]. Therefore, CD39 blockade may be an effective way to inhibit immunogenic ATP hydrolysis and prevent tumor immune escape [20].

DCs are the main antigen-presenting cells of the body, and the main precursor cells of human DCs are monocytes [21]. The activated DCs can capture tumor antigens released by tumor cells, transfer these antigens, and cross-present them to T cells [22]. The tumor antigen presentation of DCs determines the production of protective anti-tumor immunity [23]. Studies have shown that apoptosis of hepatocytes and colorectal cancer cells induced by chemotherapy or radiotherapy promotes the maturation and antigen presentation of DCs [24], and the migration ability of DCs is positively related to the concentration of eATP [25]. DCs engulf dying tumor cells to obtain and present tumor antigens to CD8^+^ T cells. Therefore, DCs play a key role in tumor antigen presentation and T cell activation.

SOX2 is a high mobility group (HMG) domain protein containing two nuclear localization signals (NLS), a dichotomous NLS motif and a basic cluster NLS motif, which are conserved in the HMG domain of transcription factors [26]. SOX2 plays a key role in maintaining stemness, and participates in cell proliferation, growth, migration, and chemotherapy resistance [27]. It is highly immunogenic, which has been confirmed in patients with lung cancer and GBM [28,29]. Moreover, the activation of SOX2-specific CD8^+^ T lymphocytes was shown after immunization with DCs primed with apoptotic CSCs [30]. Therefore, we suspect that the SOX2 transcription factor may be involved in the regulation of eATP.

Although the role of eATP inhibition by CD39 has been widely studied in the tumor microenvironment, its effects on GSCs have not yet been defined. CD39 has a key role in the initiation and regulation of DCs-mediated antigen-specific immune responses. However, it has been restricted to the observation of the eATP-mediated DCs activation. In this study, we systematically investigated the impact of CD39 on eATP level, dendritic cell recruitment, and T cell killing effect. We also discovered the molecular mechanism in which SOX2 binds to the CD39 promoter to regulate eATP level, and evaluated the enhanced immune response by inhibiting CD39 after ADM treatment in a mouse glioma model. Our study demonstrates an effective therapeutic strategy on targeting CD39 for GSCs damage and GBM therapy.

## 2. Materials and Methods

### 2.1. Cell Culture

The human glioma cell lines U251 and U87 were purchased from the Shanghai Institutes for Biological Sciences. The murine glioma cell line GL261 was obtained from American Type Culture Collection (ATCC). The human glioma cell line SHG140 was cultured from tumor tissues of patients with glioma in the First Affiliated Hospital of Soochow University [31]. The human GSC line 51A and matched non-GSC 51B were from GBM patients, as gifts from Professor Yihong Zhou at the UC Irvine Brain Tumor Research Laboratory (Irvine, CA, USA) [32]. Glioma cells were cultured in a high-glycemic DMEM with 10% fetal bovine serum (FBS), the medium was changed every 2 days, and GSCs were cultured in serum-free DMEM/F12 medium and added 2% B27 Neuro Mix (Invitrogen, Carlsbad, CA, USA), 20 ng/mL epidermal growth factor (EGF), and 10 ng/mL basic fibroblast growth factor (bFGF). Glioma cells were cultured in stem cell medium to produce GSCs, and GSCs were cultured DMEM with 10% FBS to produce differentiated glioma cells for 1 month, and these cells were used after identification as previously described [32].

### 2.2. DCs and T Cells Culture In Vitro

DCs were isolated from fresh peripheral blood mononuclear cells (PBMCs) collected from healthy volunteers (*n* = 6). First, we used Ficoll to perform gradient centrifugation on the collected peripheral blood to obtain monocytes, then magnetic beads (MiltenyiBiotec, Germany) to sort live cells were used to collect CD14^+^ monocytes. The obtained cells are fed with 10% FBS, 100 ng/mL Granulocyte-Macrophage Colony Stimulating Factor (GM-CSF) (PeproTech, Cranbury, NJ, USA) and 40 ng/mL interleukin 4 (IL-4) (PeproTech) in RPMI 1640, and 50% medium was replaced every 2 days. Tumor necrosis Factor-a (TNF-α) (PeproTech) was added on the 6^th^ day. Mature DCs were obtained on day 8–9. DCs maturation markers (CD80, CD83, CD86, HLA-DR) were analyzed by flow cytometry [33,34], IL-12 was analyzed by enzyme-linked immunosorbent assay (ELISA). For in vitro T cell culture, we used complete RPMI 1640 medium containing 10% FBS, 10 IU/mL IL-2 (PeproTech), 10 ng/mL IL-7 (PeproTech), and 10 ng/mL IL-15 (PeproTech).

### 2.3. Cytokine Assay

Released cytokine levels were determined by ELISA. IL-12p70 was quantified by the Quantikine immunoassay (R&D Systems, Emeryville, CA, USA) according to the manufacturer’s instructions.

### 2.4. Neurosphere Forming Assay

A total of 5 × 10^5^ cells were seeded in 6-well culture plates, the size of neurosphere forming was observed by microscope at 1, 3, and 7 days. The petri dish was divided into 9 areas according to #, and 3 fields of view were selected in the middle area. The area of the neurospheres was measured by Image J (v1.8.0) software, and the radius of the neurosphere was calculated. The data was measured and calculated from 3 fields/dish and 3 neurospheres/fields.

### 2.5. Soft Agar Colony Forming Assay

A total of 2000–2500 cells were mixed with 1 mL of 0.3% soft agar in DMEM/F12 supplemented with 5% bovine serum or a mitogen supplement for neurosphere culture as detailed above, then were spread onto hardened 0.5% soft agar in the same medium (1 mL per well in four corner wells of a 6-well plate). The number of colonies per well under 10× magnification after 2 weeks was calculated according to the lower part of the 6-well plate with a camera.

### 2.6. Cytotoxicity Analysis 

A total of 5 × 10^3^ cells in 100 μL well in a 96-well plate were cultured for 6–8 h, then rinsed with PBS for 3 times. ADM and/or POM-1 were added in different concentrations. After 24 h of culture, CCK8 was added and the cells were incubated for 2 h at 37 °C. The optical density value was determined by the microplate reader at 450 nm, and the cell viability was evaluated by optical density value.

### 2.7. Western Blot

The protein sample was extracted from the cells, and the protein concentration was detected using the BCA kit. After the protein sample was adjusted to a consistent concentration, the loading buffer and the protein were mixed and denatured for 20 min at a high temperature of 100 °C. The protein sample was then added to the gum sample tank. Electrophoresis was carried out at a constant voltage for about 90 min, and then the membrane treatment was carried out at a constant current for about 60–80 min. Immediately after the transferred membrane was sealed with 5% fat-free milk, the primary antibody was incubated overnight at 4 °C. After incubation with the secondary antibody for 2 h, the membrane was exposed and observed on the second day. All experiments were repeated 3 times. The protein expression was measured by Image J software and normalized with the protein level of the same set of internal reference.

### 2.8. Immunofluorescence Staining

For immunofluorescence, 50 uL of stem cell suspension was placed on an adhered glass slide. Cells were fixed with 4% paraformaldehyde at room temperature for 15 min, then the slide was air-dried, sealed with 5% BSA, placed in a humidified box and incubated with primary antibodies at 4 °C overnight. GSCs were incubated with the fluorescent secondary antibody for 1 h in a dark environment, and the slides were mounted with DAPI. Fluorescence pictures were captured through a fluorescence microscope or a confocal microscope.

### 2.9. ATP Assay

For eATP detection, cells were treated with ADM and/or POM-1 [35], then cell supernatants were collected at 1, 2, 4, 8, and 12 h after treatment, and tested by enhanced ATP analysis kit (Beyotime, Shanghai, China).

### 2.10. siRNA Design and Cell Transfection

Cells were transfected with small interfering RNA (siRNA) or CD39 overexpressing plasmid (pcDNA-CD39) in 6-well plates by use of lipofectamine 3000. The targeting siRNA sequences were designed and synthesized as follows: SOX2-siRNA: 5′-CUGCAGUCAACUCCAUGAUU-3′; CD39-siRNA: 5′-GGGCAAAUUCAGUCAGAAATT-3’.

### 2.11. Co-Cultivation System Establishment

#### 2.11.1. Co-Culture of DCs with Drug-Treated GSCs

After 24 h treatment with ADM or/and POM-1 or/and siRNA, GSCs were marked by Far Red Cell Proliferation Kit (Invitrogen, USA), and DCs were marked by CFSE Cell Proliferation Kit (Invitrogen). After 5 × 10^5^ GSCs extensive washes, GSCs were co-cultured with DCs in a 1:2 ratio in RPMI 1640 supplemented with GM-CSF (100 ng/mL), IL-4 (40 ng/mL) for 12 h. As a control, DCs were co-cultured with PBS or ADM treated GSCs for 12 h. Co-cultures of drug-treated GSCs and DCs were separately in 0.4 μm pore size filter Transwell inserts in 24-well culture plates (Corning Costar, Corning, NY, USA) [36].

#### 2.11.2. Co-Culture of T Cells with DCs and Drug-Treated GSCs

GSCs were treated in different groups for 24 h. After labeling GSCs with the CFSE cell proliferation kit, GSCs and mature DCs were co-cultured in a 24-well culture plate at a ratio of 2:1 for 12 h, T cells were seeded to the GSCs and DCs co-culture wells at a ratio of 1:3 and left to stand for 4 h. The cells were collected and stained with PI cell apoptosis detection kit (BD). Flow cytometry was used to detect the killing efficiency of T cells. 

### 2.12. Flow Cytometry

To determine the maturity of DCs, 5 × 10^5^ cells were stained with FITC-conjugated anti-HLA-DR (eBioscience, San Diego, CA, USA), CD80 (eBioscience), CD83 (eBioscience), CD86 (eBioscience), stained on ice for 30 min, washed with PBS 3 times, and then detected by flow cytometry (BD FACScan). Flow cytometry was used for detection of the DCs engulfment on GSCs after 12 h co-culture. GSCs were labeled by FarRed, DCs were labeled by CFSE, and double-positive cells were DCs phagocytosing GSCs. The recruitment efficiency of DCs was the number of double positive cells/the number of FarRed positive cells. Flow cytometry was also used for detection of the T cell killing efficiency. CFSE-positive cells were GSCs, PI-positive cells were dead cells, and double-positive cells were GSCs killed by T cells, and the killing efficiency was the number of double-positive cells/the number of CFSE-positive cells. Cells were acquired on Beckman Coulter FC500 and analyzed with CXP analysis software 2.3 (Beckman Coulter Inc, Fullerton, CA, USA). For each tube, at least 10,000 events were collected in a gate created around the viable cell population. Data were presented as percentage of cells in the GSCs cells population.

### 2.13. CD39 Promoter Plasmid Construction and Luciferase Assay

For the CD39 promoter plasmid construction, CD39 promoter region containing 1000 bp fragment was amplified by PCR from human genomic DNA using the forward and reverse primers. Then, the PCR production was cloned to pGL3.0 control plasmid between XhoI and HindIII sites. U251S and 51A GSCs grouped (PBS, SOX2siRNA) and plated in 35-mm dish 24h before transfection, followed by transfection of CD39 promoter bearing plasmid. pRL-TK was always co-transfected as the internal control. After transfection for 24h, the cells were harvested, lysed, and centrifuged for further luciferase activity analysis using Dual-Luciferase analysis system (Promega, Madison, WI, USA) according to the manufacturer’s instructions.

### 2.14. Chromatin Immunoprecipitation (ChIP) and Real-Time Reverse Transcription-Polymerase Chain Reaction (RT-PCR)

GSCs 51A and U251s were transfected with SOX2-siRNA, and ChIP was performed according to the instructions of producer (Cell signaling, Boston, MA, USA), DNA and protein in the cells were cross-linked by 37% formaldehyde, and the DNA was digested into 200–1000 bp fragments by micrococcal nuclease. Bioruptor (Diagenode, Liège, Belgium) was used to destroy the nuclear membrane. Adjustment of the cell crusher parameters as follows: amplitude 30%, pulse 5 s and stop 20 s, time 2 min. Chromatin was precipitated with anti-histone H3, anti-normal rabbit IgG and anti-SOX2 with ChIP-Grade Protein G Agarose Beads. Each sample was repeated 3 times. PCR primer sequence of CD39 was the following: sense: 5′-TACACCACTGCATTCCAGCTT-3′, antisense: 5′-GGAGATGAATTCTGCCAGAGC3′. The position of the PCR sequence was between -542 and -344 before the transcription start site. The input% of each sample was calculated according to the *Ct* value of each sample of qPCR assay (Δ*Ct*). Input percentage = 2% × 2ˆ (C[T] 2% input sample−C[T] IP sample), C[T] = *Ct* = Threshold period of PCR reaction.

### 2.15. Immunohistochemistry

The tumor tissue was fixed by paraformaldehyde and embedded in paraffin. A 4 μm tumor section was cut from the embedded tissue and incubated with specific primary anti-mouse CD4 (Cell Signaling), anti-mouse CD8 (Cell Signaling), and anti-mouse CD11b (Cell Signaling) antibodies in a humidity chamber at 4 °C overnight. Then the sections were incubated with a biotin-labeled secondary antibody for 1 h at room temperature, and finally the sections were developed with 3,3’-diaminobenzidine substrate and observed under an optical microscope.

### 2.16. Animal Experiment

The breeding and disposal of C57BL/6J mice were approved by the Animal Experiment Ethics Committee of Soochow University. GL261 cells were transfected with a luciferase-encoding lentivirus (GeneChem, Shanghai, China) and stereotactically injected into the brain of female mice (8–10 μL). After 7 days, the mice tumor model was evaluated and randomly divided into 4 groups (*n* = 6). The mice in the first group were injected intraperitoneally with DMSO (7–10 days, 14–17 days, 21–24 days) after cell inoculation. The mice in the second group were intraperitoneally injected with 6 mg/kg of ADM (7–10 days, 14–17 days, 21–24 days) [37], the mice in the third group were injected intraperitoneally with 5 mg/kg POM-1 (7–10 days, 14–17 days, 21–24 days) [38], and the mice in the fourth group were injected intraperitoneally with ADM and POM-1 (7–10 days, 14–17 days, 21–24 days). Mice intracranial tumors were imaged using IVIS spectral real-time imaging system (Perkinelmer, Waltham, Massachusetts, USA) at day 7, 14, and 28. The mice were sacrificed 6 weeks after tumor implantation for immunohistochemistry research.

### 2.17. Statistical Analysis

All data were analyzed using GraphPad Prism 8.0 (GraphPad Software Inc, San Diego, CA, USA). Most of the experimental data were statistically analyzed using Student’s *t*-test, and the data of two or more groups and differences between groups were evaluated using one-way analysis of variance (ANOVA). The survival rate of mice was evaluated by Kaplan–Meier method and analyzed by log-rank test. The significance of the *p*-value is NSP > 0.05, * *p* < 0.05, ** *p* < 0.01, *** *p* < 0.001, **** *p* < 0.0001.

## 3. Results

### 3.1. The Difference of CD39 mRNA Was Expressed in Glioma Histological Subtype and GBM Grade

**Figure 1:**
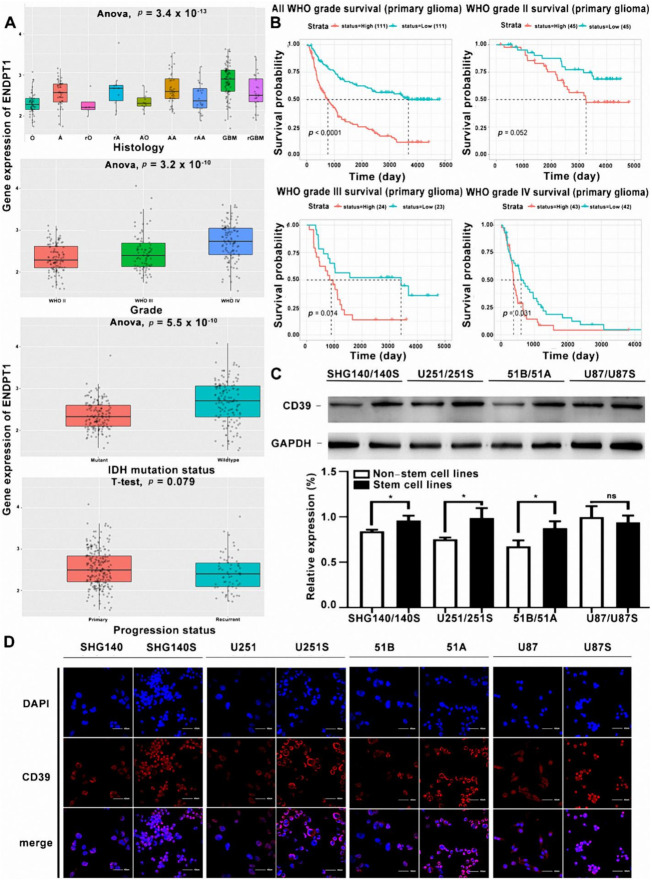
The expression of CD39 mRNA in the histological subtypes of glioma and GBM grade is negatively related with the overall survival (OS) of patients with glioma. In the Chinese Glioma Genome Atlas (CGGA), we extracted 325 cases of glioma patients, first, the expression of CD39 mRNA in different histological subtypes was analyzed. Histological subtypes included astrocytoma (**A**), anaplastic oligodendroglioma (AO), oligodendroglioma (O), anaplastic oligoastrocytoma (AOA), oligoastrocytoma (OA), anaplastic astrocytoma (AA), GBM, and recurrence of histological subtypes. (**A**) The expression of CD39 mRNA in different histological subtypes. (**B**) The interrelation of CD39 expression and the overall survival of patients with primary glioma in the CGGA equation data set. (**C**) Western blot analysis of CD39 protein expression in GSCs and matched non-GSCs. Uncropped Western Blot was shown in Figure S4. Data are represented as the mean ± s.d., *n* = 3, NS. not significant, *p >* 0.05, * *p* < 0.05, student’s *t*-test. Uncropped Western Blots was shown in Figure S4. (**D**) Immunofluorescence analysis of CD39 protein expression in GSCs and matched non-GSCs. Scale bar = 40 μm.

The expression of CD39 was found highest in GBM among all subtypes of different histological glioma. Furthermore, the expression of CD39 mRNA is the highest in grade IV of the WHO classification of glioma. Moreover, in the different genotyping of CD39, the expression in the wild subtype (original gene) was higher than the mutant subtype (gene mutation), and the CD39 expression in primary gliomas were higher than recurrent gliomas (Figure 1A).

### 3.2. CD39 mRNA Was Negatively Related with Overall Survival (OS) of Glioma Patients

To further clarify whether the high expression of CD39 in glioma was related to the prognosis of patients, we analyzed the relationship between the expression of CD39 and the overall survival of patients with primary glioma by CGGA equation datasets. The results showed that the survival time of the low CD39 expression group was significantly higher than that of the high expression group, especially in grade Ⅲ and Ⅳ glioma patients (Figure 1B).

### 3.3. CD39 Expression Was Upregulated in GSCs

To identify the effect of CD39 on the characteristics of GSCs, we first detected the expression of stem cell characteristic markers in GSCs and matched non-GSC in 4 cell lines (Figure S1A). Then we analyzed the expression of CD39 protein in these cell lines. Interestingly, western blot showed that CD39 protein was significantly up-regulated in GSCs comparing to matched non-GSC, except for U87 cell lines (Figure 1C and Figure S4), and consistent results were found by immunofluorescence staining (Figure 1D).

### 3.4. The eATP Concentration of GSC Is Lower Compared with Non-GSC

Previous studies have shown that the release of ATP in tumor cells depends on tumor stress and CD39 expression [13]. Based on the fact that GSCs are resistant on chemotherapy, we speculated a different ATP release between GSCs and non-GSCs in the TME after chemotherapy. ADM is a known immunogenic chemotherapy that can induce the release of ATP [14]. Therefore, we used ADM to induce GSCs to release ATP.

**Figure 2:**
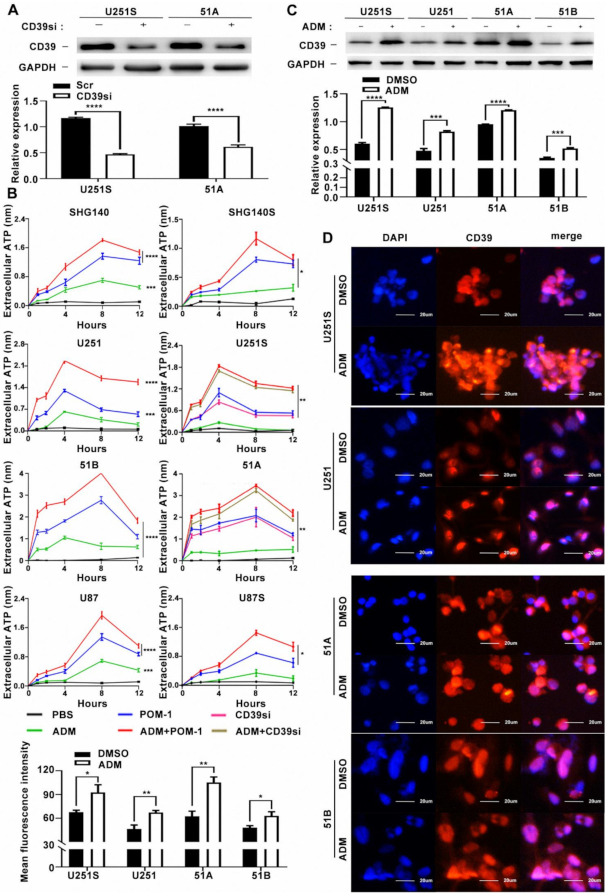
The expression of CD39 is upregulated after ADM treatment in GSCs and matched non-GSCs. (**A**) Western blot analysis of CD39 protein knockdown. Uncropped Western Blot was shown in Figure S4. (**B**) GSCs were treated with PBS, POM-1 (100 μM), ADM (the drug concentration is the IC50 of the cell lines) and POM-1 (100 μM) + ADM (IC50). The concentration of eATP in the culture supernatant of different treatment groups for 1, 2, 4, 8 and 12 h. Western blot (**C**) and immunofluorescence (**D**) analysis of CD39 protein expression in GSCs and matched non-GSCs after AMD treatment. Uncropped Western Blot was shown in Figure S4. Scale bar = 20 μm. Data in B are represented as the mean ± s.d., *n* = 3, BLANK *p >* 0.05, * *p* < 0.05, ** *p* < 0.01, likelihood ratio test. Data in A, C, D are represented as the mean ± s.d., *n* = 3, *** *p* < 0.001, **** *p* < 0.0001, Student’s *t*-test.

The cell counting kit 8 (CCK8) is used to assess the survival rate of GSC and non-GSC. We applied different concentration gradients of ADM to treat non-GSCs (51B, U251, SHG140, U87) and matched GSCs (51A, U251S, SHG140S, U87S), and the IC50 (24H) results of each cell line showed 0.48 μM for U251, 1.87 μM for U251S, 0.42 μM for 51B, 0.48 μM for SHG140, 2.21 μM for SHG140S, 0.116 μM for U87 and 1.542 μM, for U87S (Figure S1). These results suggested that GSCs require a higher concentration than non-GSCs on ADM intervention. We treated each cell line with ADM IC50 concentration for 24 h to induce tumor cells to release ATP. In order to detect the relationship between the expression of CD39 on the cell membrane surface and the level of extracellular ATP, we used CD39 inhibitor POM-1 and CD39 siRNA to knockdown. The expression after CD39 knockdown is shown in Figure 2A and Figure S4.

No significant ATP release was detected after ADM treatment in glioma cells. However, POM-1 treatment or CD39-siRNA transfection increased the concentration of eATP. Interestingly, the concentration of eATP in the ADM and POM-1 or CD39-siRNA combination-treated cells was significantly increased compared to ADM, POM-1 or CD39-siRNA treated cells alone. We found that the concentration of eATP was higher in non-stem glioma cells than GSCs (Figure 2B). It is well-known that most of the eATP released by apoptotic cell were hydrolyzed and decreased by CD39. In our study, CD39 inhibition increased the concentration of eATP, suggesting a crucial role of CD39 in regulating the eATP in GSCs.

### 3.5. CD39 Is Overexpressed More Significantly in GSCs after ADM Treatment

To further determine the expression of CD39 after ADM treatment, we treated U251S, U251, 51A, 51B with ADM 24 h (used as IC50 concentration). The expression of CD39 is shown in Figure 2C and Figure S4. Interestingly, CD39 expression in both stem cells and non-stem cells was upregulated after the ADM treatment, especially in stem cells. The immunofluorescence experiment also obtained similar results (Figure 2D).

### 3.6. CD39 Positively Regulates the Self-Renewal, Proliferation and Stemness of GSCs In Vitro

**Figure 3:**
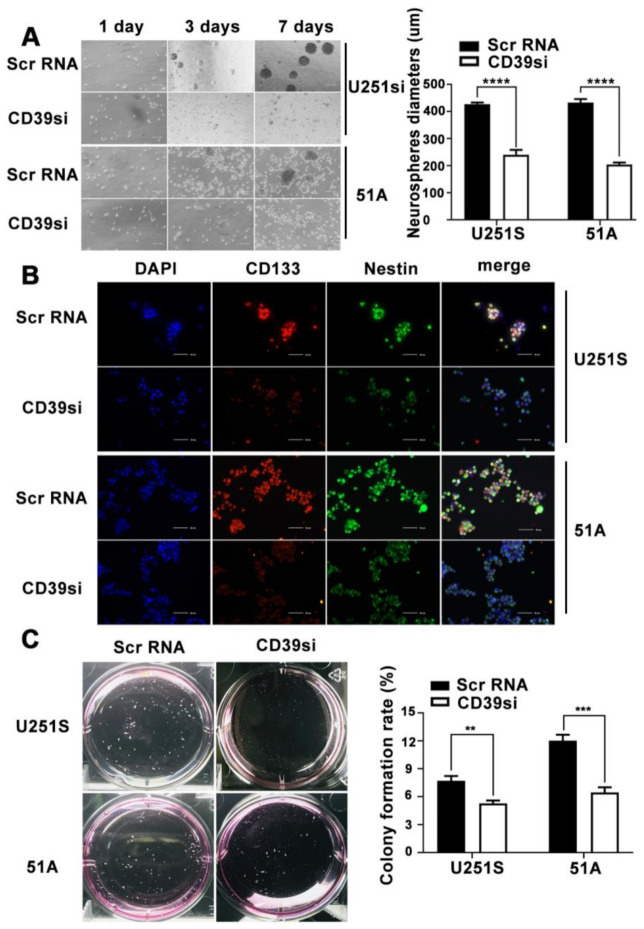
Effect of CD39 on the self-renewal, proliferation, and stemness of GSCs in vitro. **(A**) The representative images of GSCs neurospheres showed that the neurosphere formation ability of GSCs was significantly inhibited by CD39 siRNA treatment. (**B**) Immunofluorescence staining of GSCs transfected with CD39 siRNA. Scale bar = 40 μm. (**C**) Colony Forming Assay showing the proliferation ability of GSCs. Data in A, C are represented as the mean ± s.d., *n* = 3, ** *p* < 0.01, *** *p* < 0.001, **** *p* < 0.0001, Student’s *t*-test.

Neurosphere formation, fluorescence on stemness marker, and soft agar colony forming assay were used to clarify whether CD39 regulates the GSCs self-renewal, proliferation and stemness. When CD39 was knockdown in U251S and 51A cells, we confirmed the decrease of forming neurosphere ability by measuring and quantifying the diameter (Figure 3A), and immunofluorescence also indicated downregulation of the stem markers CD133 and Nestin (Figure 3B). Moreover, the expression of CD39 is positively related with the number of GSCs colonies (Figure 3C). In addition, the number of GSCs colonies was also significantly reduced.

### 3.7. Combination of ADM and CD39 Blockade in GSCs Induced the Increase of DCs Phagocytosis and T Cells Lysis

**Figure 4:**
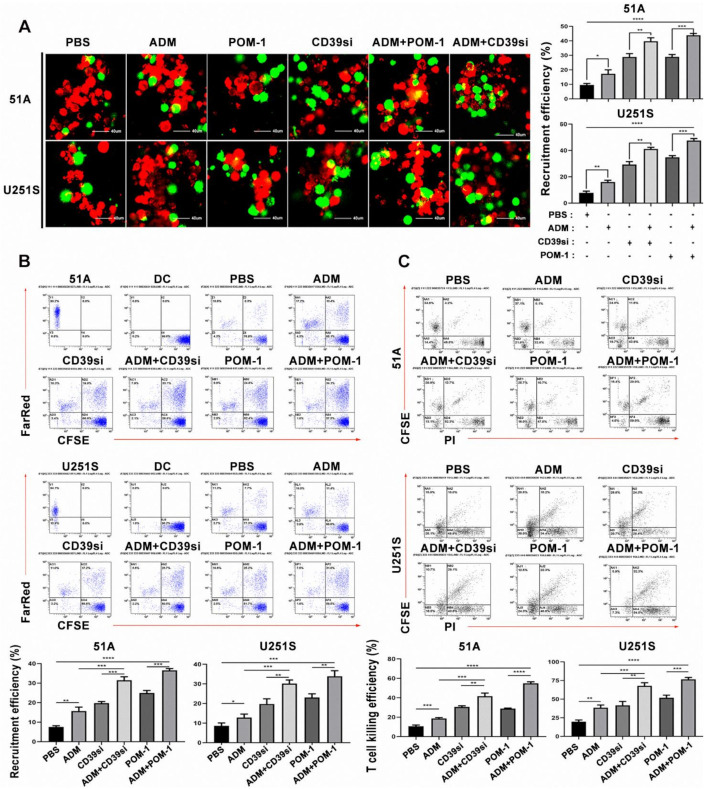
The recruitment and phagocytosis of DCs on GSCs and the killing effect of T cells were related to the level of eATP release. GSCs were treated with DMSO, POM-1 (100 μM), CD39-siRNA transfection and/or ADM (IC50). (**A**) The recruitment and phagocytosis of DCs to GSCs were captured by confocal microscope after co-culture for 12 h. Scale bar = 40 μm. DCs were labeled by CFSE (green), and GSCs were labeled by Far Red (red). (**B**) Flow cytometry was used to analyze the phagocytosis of DCs at 12 h. (**C**) Flow cytometry was used to analyze the killing efficiency of T cells at 12 h. Data in A, B, C were represented as the mean ± s.d., *n* = 3, * *p* < 0.05, ** *p* < 0.01, *** *p* < 0.001, **** *p* < 0.0001, Student’s *t*-test.

We first used PBMCs to induce mature DCs (Figure S2A), which were showed by CD80, CD83, CD86 and HIL-DR increase using flow cytometry analysis (Figure S2B). The concentration of IL-12 released by dendritic cells increased significantly (Figure S2C). In order to study phagocytosis of DCs on GSCs, mature DCs were co-cultured with GSCs and matched non-GSCs, we found that DCs approached GSCs at 2 h and gradually swallowed GSCs at 12 h (Figure S3A). DCs were co-cultured with GSCs or non-GSCs treated with ADM. Compared with GSCs, non-GSCs recruited more DCs (Figure S3B). DCs were co-cultured with GSCs treated with ADM, POM-1 or transfected with CD39-siRNA. Compared with the control group, the recruitment and phagocytosis of DCs was enhanced by CD39 inhibition or knockdown, and the effect was further increased in combination of ADM treatment and CD39 inhibition or knockdown using fluorescence microscope observation (Figure 4A) and flow cytometry analysis (Figure 4B). We found a consistent trend in the killing efficiency of T cells (Figure 4C). These data suggested that combination ADM and CD39 inhibition increased eATP concentration around GSCs, which recruited DCs to phagocytose damaged GSCs and activated T cells to kill target cells.

### 3.8. DCs Phagocytosis on Target Cells Was Promoted by SOX2 Knockdown in GSCs

**Figure 5:**
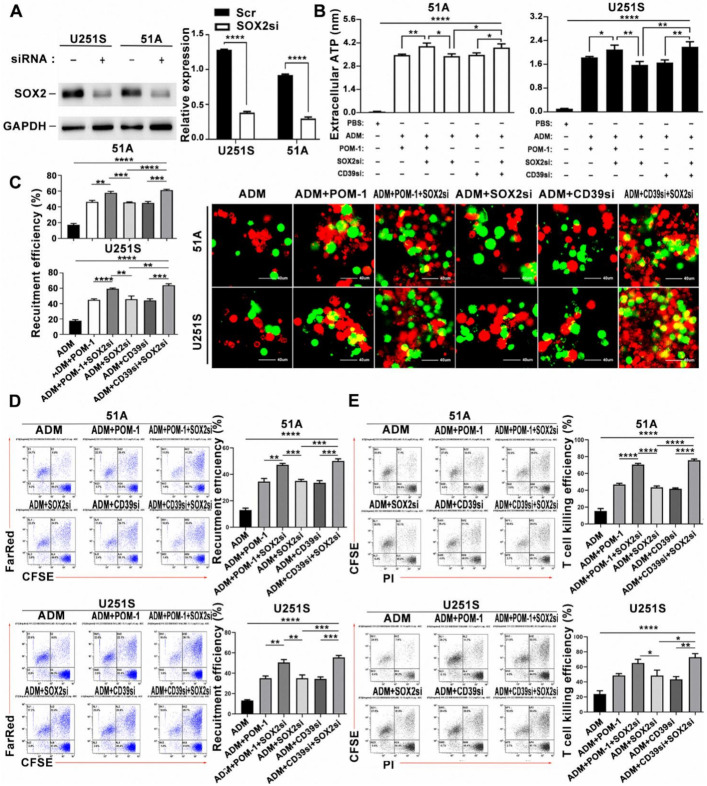
SOX2 downregulation increased the functions of DCs and T lymphocytes. (**A**) Western blot analysis of SOX2 protein knockdown. Uncropped Western Blot was shown in Figure S4. (**B**) The concentration of eATP in the culture supernatant of different treatments was detected. Confocal microscope (**C**) and flow cytometry (**D**) were used to analyze the phagocytosis of GSCs by DCs after 12 h of different treatments. DCs were labeled by CFSE (green), and GSCs were labeled by Far Red (red). Scale bar = 40 μm. (**E**) Flow cytometry was used to analyze the killing efficiency of T cells on GSCs after 12 h of different treatments. Data in A, B, C, D, E were represented as the mean ± s.d., *n* = 3, * *p* < 0.05, ** *p* < 0.01, *** *p* < 0.001, **** *p* < 0.0001, Student’s *t*-test.

To explore the mechanism of CD39 up-regulation in GSCs, TIMER analysis for immuno-infiltrated cells was performed according to the expression of SOX2 in glioma patients. We found that SOX2 was negatively related with the infiltration of myeloid DCs and plasma DCs (Figure S3C). This is the same as the increasing trend of dendritic cell recruitment efficiency in the patients of CD39 low expression. Thence, we used siRNA to knockdown SOX2 expression, and the knockdown efficiency was detected by western blot (Figure 5A and Figure S4). Then we treated GSCs with CD39 siRNA and SOX2 siRNA, and co-cultured GSCs with DCs. As we expected, the recruitment efficiency of DCs increased in both the CD39si and the SOX2si group (Figure S3D,E). Next, we measured eATP in different groups. Compared with the control group, eATP concentration was increased after SOX2 knockdown, which was basically the same as the CD39 interference group. Moreover, eATP was further increased in combination of ADM, CD39 inhibition and SOX2 knockdown (Figure 5B). GSCs were co-cultured with DCs alone or DCs and T cells together. The phagocytosis of DCs was observed by confocal microscope (Figure 5C) and flow cytometry (Figure 5D). Flow cytometry analyzed the killing efficiency of T cells (Figure 5E). The recruitment and phagocytic ability of DCs and the killing efficiency of T cells were increased when SOX2 was knockdown. These results suggested an enhanced effect of SOX2 knockdown in ATP release and DCs phagocytosis in GSCs.

### 3.9. SOX2 Binding to the CD39 Promoter Region

**Figure 6:**
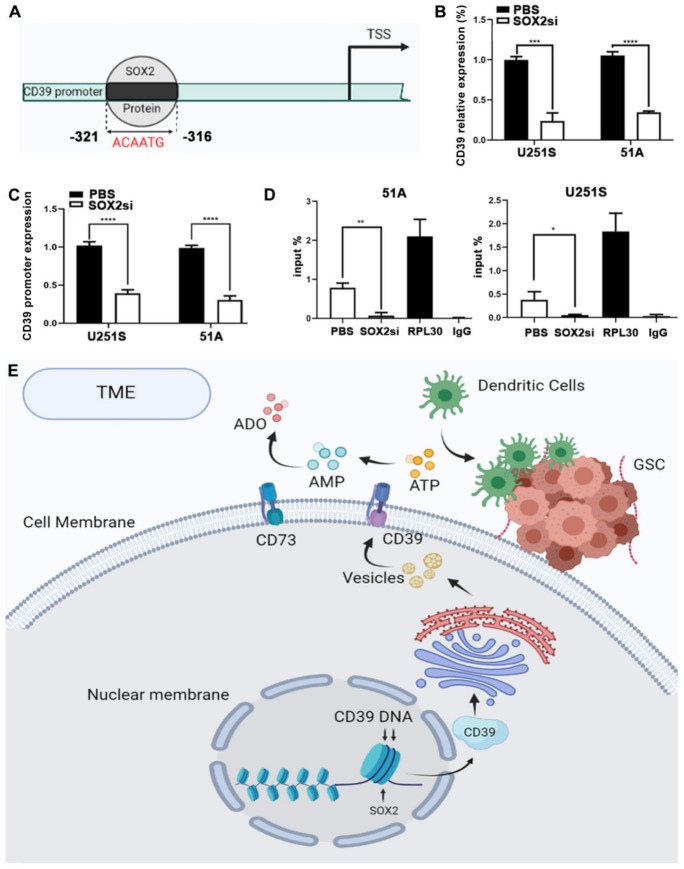
SOX2 bound to the CD39 gene promoter region to regulate eATP concentration and further affected immune cells function. (**A**) Schematic diagram of the binding sequence of SOX2 protein and CD39 promoter. (**B**) U251S and 51A GSCs transfected with CD39 promoter luciferase reporter gene and Renilla luciferase internal control were used to detect the binding of SOX2 and CD39 promotor, and luciferase activity was normalized to Renilla activity. (**C**) QPCR analysis of the expression of CD39 after SOX2 knockdown. (**D**) ChIP and RT-qPCR analysis for SOX2 protein binding to CD39 gene promoter region. (**E**) The mechanism diagram describes the process of SOX2 regulating the CD39 promoter. Data in B, C, D are represented as the mean ± s.d., *n* = 3, * *p* < 0.05, ** *p* < 0.01, *** *p* < 0.001, **** *p* < 0.0001, student’s t test.

Based on the above observations, we speculate that there is a positive interrelation between CD39 and SOX2. To clarify the molecular mechanism of this interaction, ChIP was used to assess whether SOX2 binds to the CD39 promoter. The DNA of GSCs was digested into fragments of 150–1000 bp, and the region of SOX2 binding to the CD39 promotor was located at [(−321)~(−316 )] on the CD39 sequence. The binding sequence is ACAATG (Figure 6A). Then we used QPCR to verify CD39 mRNA expression after SOX2 knockdown, and we found that CD39 expression was significantly down-regulated after SOX2 knockdown (Figure 6B). In addition, we used the luciferase reporter gene to directly assess the CD39 gene promoter activity, and found that the transcription activity of the CD39 promoter region was significantly reduced after SOX2 knockdown (Figure 6C). The result of ChIP showed a decreased expression of CD39 mRNA when GSCs 51A and U251S were transfected with SOX2-siRNA (Figure 6D). These data indicate that SOX2 regulates the concentration of eATP by binding to the CD39 promoter (Figure 6E).

### 3.10. CD39 Plays an Important Role in the Immune Escape of GSCs

**Figure 7:**
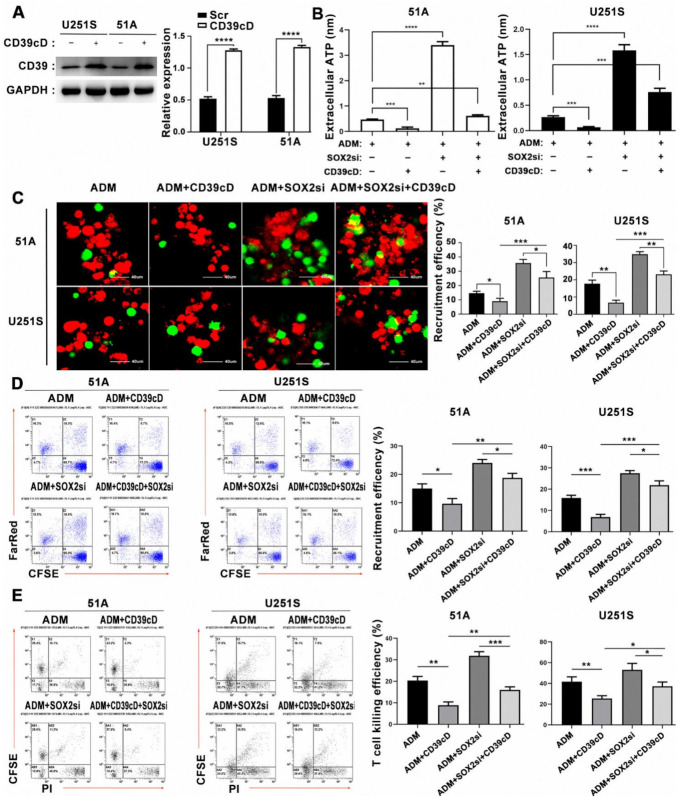
Overexpression of CD39 protein blocked DCs and T cells activation induced by SOX2 knockdown. (**A**) Western blot analysis of CD39 protein overexpression. Uncropped Western Blot was shown in Figure S4. (**B**) GSCs were transfected with SOX2 siRNA or CD39 plasmid, then the concentration of eATP in the culture supernatants was detected after 12 h of treatment with ADM (IC50). DCs were co-cultured with GSCs for 12 h, and the recruitment and phagocytosis of DCs were analyzed by confocal microscope (**C**) and flow cytometry (**D**). DCs were labeled by CFSE (green), and GSCs were labeled by Far Red (red). Scale bar = 40 μm. (**E**) The killing efficiency of T cells on GSCs was analyzed by flow cytometry. Data in A, B, C, D, E were represented as the mean ± s.d., *n* = 3, * *p* < 0.05, ** *p* < 0.01, *** *p* < 0.001, **** *p* < 0.0001, Student’s *t*-test.

51A and U251S were transfected with pcDNA-CD39 to overexpress CD39 cDNA, and the transfection efficiency was verified by western blot analysis (Figure 7A and Figure S4). As depicted in Figure 7B, SOX2 knockdown increased the eATP concentration, while the CD39 overexpression decreased the eATP concentration. Interestingly, CD39 overexpression reverted the increase of eATP concentration induced by SOX2 knockdown. The recruitment of DCs and the killing efficiency of T cells measured by flow cytometry and confocal microscopy analysis showed consistent results (Figure 7C–E), suggesting that CD39 directly regulates the eATP concentration of GSCs, and SOX2 regulates the eATP concentration through CD39. The high expression of CD39 in GSCs plays an important role in tumor evasion from immune surveillance.

### 3.11. CD39 Blockade Improves Antitumor Immunity in an Intracranial Glioma Model

**Figure 8:**
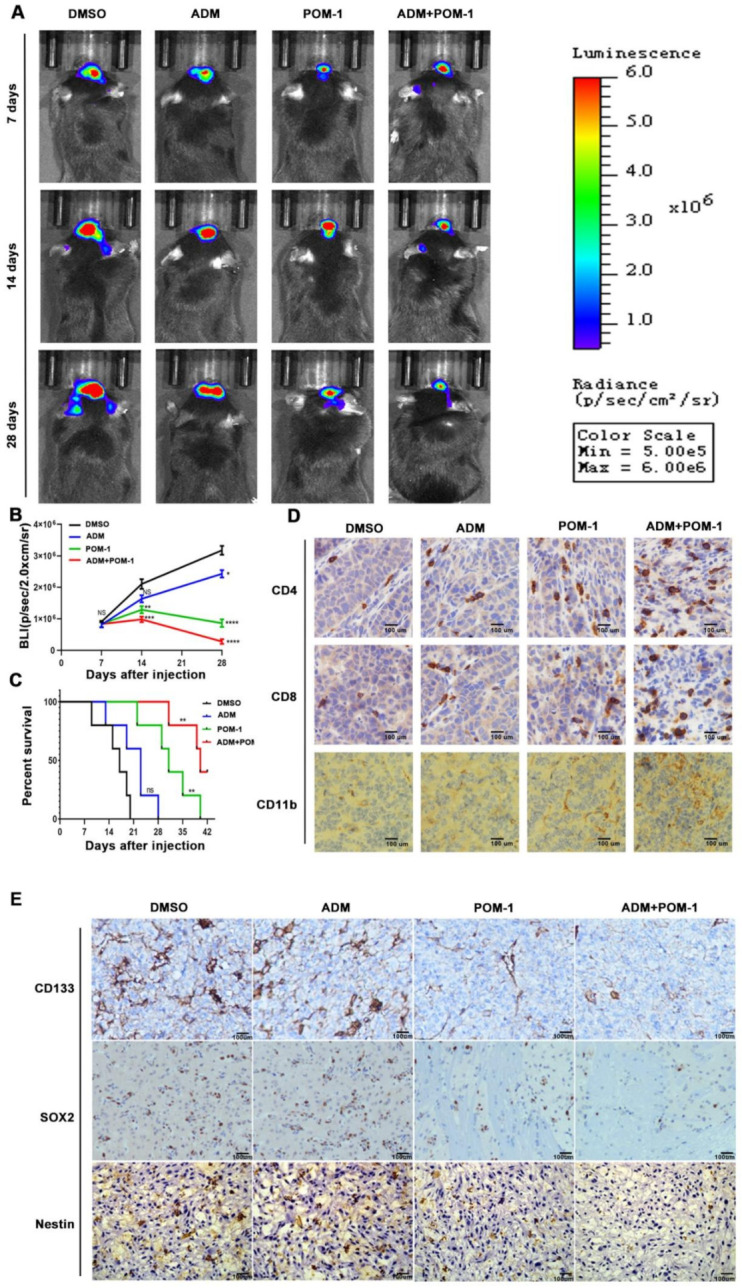
The combined application of POM-1 and ADM upregulated the protective tumor immunity and reduced the number of GSCs in vivo. (**A**) The in vivo imaging system (IVIS) showed the photon measurements around the tumor area on days 7, 14, and 28. (**B**) Quantitative analysis of bioluminescence images. Data are shown as the mean ± s.d., *n* = 5, NS*P>*.05, * *p* < 0.05, ** *p* < 0.01, *** *p* < 0.001, **** *p* < 0.0001, vs. control, Student’s *t*-test. (**C**) The overall survival time of mice in different treatment groups was evaluated by Kaplan–Meier method. Data are shown as mean ± SD, n = 5, NSP > 0.05, ** *p* < 0.01, vs. control, log-rank test. (**D**) The paraffin-embedded tumor tissues were used for pathology and IHC analysis. Tumor sections were stained for anti-mouse CD4, CD8, CD11b antibodies. (**E**) Sections were stained for anti-mouse CD133, SOX2 and Nestin antibodies. The images were captured with an optical microscope (×400). Scale bar corresponds to 100 μm.

To further explore the anti-tumor effect on ADM and CD39 blockade in vivo, C57BL/6J mice were intracranially implanted with GL261s GSCs transfected with luciferase encoding lentivirus. The tumor size at 7, 14, 28 days after cells implantation was evaluated by the in vivo imaging system. The photon measurement for tumor size showed that POM-1 inhibited tumor growth (Figure 8A,B), and the survival time of mice treated with POM-1 was significantly longer than that of control group (Figure 8C). The effects on tumor growth inhibition and survival time extension were more obvious in combination of ADM and POM-1 treatment compared with ADM or POM-1 alone. Immunohistochemistry analysis showed that CD4+, CD8+ T cells and CD11b+ DCs were significantly increased in the tumors in the POM-1 group, especially POM-1 combined with ADM group (Figure 8D). Meanwhile, stem cell components of the tumors were significantly reduced in the POM-1 group, especially POM-1 combined with ADM group (Figure 8E). These data indicated that CD39 inhibition improves anti-tumor immunity in a mouse glioma model.

## 4. Discussion

An immunosuppressive environment promotes tumor spread, and primary or acquired drug resistance is common in cancer immunotherapy [39]. In this study, we demonstrated the effect of CD39 on increasing eATP produced by chemotherapy in GSCs, indicating that CD39 blockade is an effective strategy to enhance anti-tumor immunity.

Studies have shown that the expression of CD39 is increased in pancreatic cancer, rectal cancer primary tumors and metastatic tumors, and that CD39 blockade provides better prognostic value [40,41]. We confirmed here that CD39 is highly expressed in glioma stem cells compared to glioma non-stem cells. Chemotherapy drugs, such as oxaliplatin and mitoxantone, induce cell apoptosis and ATP release. In CD39^+^ cancer cells, they cannot cause an effective immunogenic response due to eATP hydrolysis by CD39. This view was also confirmed in our study. The level of eATP levels showed no significant changes after ADM treatment in GSCs, however, it was changed notably in glioma cells. The eATP level of GSCs treated with CD39 inhibition, especially the combination of CD39 inhibition and ADM, was significantly increased. The enzyme NPP1 or CD38 produces AMP, and it leads to the production of Ado in the tumor microenvironment through other means, when CD39 activity is lacking [42]. In this case, it is meaningful for us to study the effect of CD39. We performed a knockdown of CD39 using siRNA in GSCs, which inhibits the expression of CD39 on the cell membrane surface more effectively than POM-1. The experimental results were consistent with our expectations. When GSCs were transfected with CD39 siRNA and CD39 siRNA combined with ADM treatment, eATP levels were upregulated. However, when GSCs were transfected with overexpressed CD39 cDNA and treated with ADM and SOX2 inhibition, eATP level was significantly decreased, which suggests that CD39 is unique in the ATP/adenosine cascade. These data offer theoretical potential to influence anti-tumor immunity in two ways. On one hand, more pro-inflammatory and pro-proliferative danger signals are produced in TME by preventing the phosphoric hydrolysis of ATP. On the other hand, blocking downstream accumulation of extracellular adenosine reverses direct adenosine receptor-mediated immunosuppression and long-term established immunosuppressive TME [43].

One mechanism of tumor escape is that the suppression of the immune response is achieved in part by ATP and adenosine accumulation in TME [18]. Low eATP level promotes tumor proliferation and immunosuppression, while high ATP level preferentially activates infiltrating DCs [44]. DCs process and present tumor-associated antigens (TAAs), thereby allowing simultaneous stimulation of helper T lymphocytes and cytotoxic T lymphocytes, minimizing immune escape. Therefore, DCs play a key role in the process of anti-tumor immunity. In this article, we co-cultured DCs, T cells, and GSCs treated with different methods. The results proved that the recruitment and phagocytosis of DCs and the T cell killing effect on GSCs are positively related with eATP levels. Therefore, GSCs upregulate eATP levels through the expression of CD39, which inhibits the recruitment and antigen presentation of DCs and induces immune escape.

The transcription factor SOX2 plays a key role in various adult stem cell populations [26]. In addition to binding to a specific DNA consensus sequence, the core HMG domain of SOX2 also contains a nuclear localization and a nuclear export signal. The function of the C-terminal transactivation domain is to recognize and bind to the promoter of the target gene, thereby activating or inhibiting gene expression [45]. Our study found that SOX2 binds to the CD39 promoter region, while SOX2 knockdown elevates eATP levels, and increases the recruitment and phagocytosis of DCs, suggesting that SOX2 inhibits the production of eATP by CD39 upregulation.

Our in vivo data also showed that treatment of mice with POM-1 combined with ADM reduced the tumor size of GSCs-transplanted mice, prolonged the survival period, and increased immune cell infiltration. Similarly, the mouse model produced positive effects on the immune system using the monoclonal antibody IPH5201, which blocked CD39, with encouraging results. [46]. Recently, two independent reagents for CD39 blockade performed clinical trials for the first time (NCT03884556 [47], NCT04261075 [46]).

## 5. Conclusions

In conclusion, we reported the molecular mechanism of CD39 in the immune escape of GSCs. SOX2 bound to the CD39 promoter, regulated the level of eATP in TME, and affected the recruitment of DCs to apoptotic tumor cells and T cell killing effect on GSCs, leading to immune escape. CD39 blockade increased the concentration of eATP released by ADM, and enhanced the sensitivity of ADM chemotherapy on GSCs. This discovery has important clinical implications.

## Data Availability

All data generated or analyzed during this study are included in this article and its Supplementary Material files. Further enquiries can be directed to the corresponding author.

## Supplementary Materials

The following supporting information can be downloaded at: https://www.mdpi.com/article/10.3390/cancers14030783/s1, Figure S1: GSCs presented more stem cell markers and were more resistant for chemotherapy than non-GSCs; Figure S2: PBMCs were induced into functionally mature DCs after 1 week; Figure S3: DCs gradually approach and phagocytize the GSCs, Figure S4. Full Uncropped Western Blots

## References

[1] De Souza R.M., Shaweis H., Han C., Sivasubramaniam V., Brazil L., Beaney R., Sadler G., Al-Sarraj S., Hampton T., Logan J. (2016). Has the survival of patients with glioblastoma changed over the years?. Br. J. Cancer.

[2] Greer L., Pannullo S.C., Smith A.W., Taube S., Yondorf M.Z., Parashar B., Trichter S., Nedialkova L., Sabbas A., Christos P. (2017). Accelerated Hypofractionated Radiotherapy in the Era of Concurrent Temozolomide Chemotherapy in Elderly Patients with Glioblastoma Multiforme. Cureus.

[3] Zarnett O.J., Sahgal A., Gosio J., Perry J., Berger M.S., Chang S., Das S. (2015). Treatment of elderly patients with glioblastoma: A systematic evidence-based analysis. JAMA Neurol..

[4] Wilson R.J., Thomas C.D., Fox R., Roy D.B., Kunin W.E. (2004). Spatial patterns in species distributions reveal biodiversity change. Nature.

[5] Lamour V., Henry A., Kroonen J., Nokin M.J., von M.Z., Fisher L.W., Chau T.L., Chariot A., Sanson M., Delattre J.Y. (2015). Targeting osteopontin suppresses glioblastoma stem-like cell character and tumorigenicity in vivo. Int. J. Cancer.

[6] Mao D.D., Gujar A.D., Mahlokozera T., Chen I., Pan Y., Luo J., Brost T., Thompson E.A., Turski A., Leuthardt E.C. (2015). A CDC20-APC/SOX2 Signaling Axis Regulates Human Glioblastoma Stem-like Cells. Cell Rep..

[7] Roesch S., Rapp C., Dettling S., Herold-Mende C. (2018). When Immune Cells Turn Bad-Tumor-Associated Microglia/Macrophages in Glioma. Int. J. Mol. Sci..

[8] Guadagno E., Presta I., Maisano D., Donato A., Pirrone C.K., Cardillo G., Corrado S.D., Mignogna C., Mancuso T., Donato G. (2018). Role of Macrophages in Brain Tumor Growth and Progression. Int. J. Mol. Sci..

[9] Filley A.C., Henriquez M., Dey M. (2017). Recurrent glioma clinical trial, CheckMate-143: The game is not over yet. Oncotarget.

[10] Hanahan D., Weinberg R.A. (2011). Hallmarks of cancer: The next generation. Cell.

[11] Yegutkin G.G. (2008). Nucleotide- and nucleoside-converting ectoenzymes: Important modulators of purinergic signalling cascade. Biochim. Biophys. Acta.

[12] De Andrade Mello P., Coutinho-Silva R., Savio L.E.B. (2017). Multifaceted Effects of Extracellular Adenosine Triphosphate and Adenosine in the Tumor-Host Interaction and Therapeutic Perspectives. Front. Immunol..

[13] Silva-Vilches C., Ring S., Mahnke K. (2018). ATP and Its Metabolite Adenosine as Regulators of Dendritic Cell Activity. Front. Immunol..

[14] Di Virgilio F., Adinolfi E. (2017). Extracellular purines, purinergic receptors and tumor growth. Oncogene.

[15] Kroemer G., Galluzzi L., Keep O., Zitvogel L. (2013). Immunogenic cell death in cancer therapy. Annu. Rev. Immunol..

[16] Allard B., Beavis P.A., Darcy P.K., Stagg J. (2016). Immunosuppressive activities of adenosine in cancer. Curr. Opin. Pharmacol..

[17] Antonioli L., Pacher P., Vizi E.S., Haskó G. (2013). CD39 and CD73 in immunity and inflammation. Trends Mol. Med..

[18] Allard B., Longhi M.S., Robson S.C., Stagg J. (2017). The ectonucleotidases CD39 and CD73: Novel checkpoint inhibitor targets. Immunol. Rev..

[19] Sun X., Han L., Seth P., Bian S., Li L., Csizmadia E., Junger W.G., Schmelzle M., Usheva A., Tapper E.B. (2013). Disordered purinergic signaling and abnormal cellular metabolism are associated with development of liver cancer in Cd39/ENTPD1 null mice. Hepatology.

[20] Nikolova M., Carriere M., Jenabian M.A., Limou S., Younas M., Kök A., Huë S., Seddiki N., Hulin A., Delaneau O. (2011). CD39/adenosine pathway is involved in AIDS progression. PLoS Pathog..

[21] Idoyaga J., Steinman R.M. (2011). SnapShot: Dendritic Cells. Cell.

[22] Diamond M.S., Diamond M.S., Kinder M., Matsushita H., Mashayekhi M., Dunn G.P., Archambault J.M., Lee H., Arthur C.D., White J.M. (2011). Type I interferon is selectively required by dendritic cells for immune rejection of tumors. J. Exp. Med..

[23] Fuertes M.B., Kacha A.K., Kline J., Woo S.R., Kranz D.M., Murphy K.M., Gajewski T.F. (2011). Host type I IFN signals are required for antitumor CD8+ T cell responses through CD8α+ dendritic cells. J. Exp. Med..

[24] Apetoh L., Ghiringhelli F., Tesniere A., Obeid M., Ortiz C., Criollo A., Mignot G., Maiuri M.C., Ullrich E., Saulnier P. (2007). Toll-like receptor 4-dependent contribution of the immune system to anticancer chemotherapy and radiotherapy. Nat. Med..

[25] Schnurr M., Ghiringhelli F., Tesniere A., Obeid M., Ortiz C., Criollo A., Mignot G., Maiuri M.C., Ullrich E., Saulnier P. (2003). ATP gradients inhibit the migratory capacity of specific human dendritic cell types: Implications for P2Y11 receptor signaling. Blood.

[26] Novak D., Hüser L., Elton J.J., Umansky V., Altevogt P., Utikal J. (2020). SOX2 in development and cancer biology. Semin. Cancer Biol..

[27] Li J., Pan G., Cui K., Liu Y., Xu S., Pei D. (2007). A dominant-negative form of mouse SOX2 induces trophectoderm differentiation and progressive polyploidy in mouse embryonic stem cells. J. Biol. Chem..

[28] Güre A.O., Stockert E., Scanlan M.J., Keresztes R.S., Jäger D., Altorki N.K., Old L.J., Chen Y.T. (2000). Serological identification of embryonic neural proteins as highly immunogenic tumor antigens in small cell lung cancer. Proc. Natl. Acad. Sci. USA.

[29] Schmitz M., Temme A., Senner V., Ebner R., Schwind S., Stevanovic S., Wehner R., Schackert G., Schacert H.K., Fussel M. (2007). Identification of SOX2 as a novel glioma-associated antigen and potential target for T cell-based immunotherapy. Br. J. Cancer.

[30] Xu Q., Liu G., Yuan X., Xu M., Wang H., Ji J., Konda B., Black K.L., Yu J.S. (2009). Antigen-specific T-cell response from dendritic cell vaccination using cancer stem-like cell-associated antigens. Stem. Cells.

[31] Li Y., Sun T., Chen Z., Shao Y., Huang Y., Zhou Y. (2021). Characterization of a new human astrocytoma cell line SHG140: Cell proliferation, cell phenotype, karyotype, STR markers and tumorigenicity analysis. J. Cancer.

[32] Yang W., Li Y., Gao R., Xiu Z., Sun T. (2020). MHC class I dysfunction of glioma stem cells escapes from CTL-mediated immune response via activation of Wnt/β-catenin signaling pathway. Oncogene.

[33] Chometon T.Q., Siqueira M.D.S., Sant Anna J.C., Almeida M.R., Gandini M. (2020). A protocol for rapid monocyte isolation and generation of singular human monocyte-derived dendritic cells. PLoS ONE.

[34] Kreitinger J.M., Shepherd D.M. (2018). Dendritic Cell Assays. Methods Mol. Biol..

[35] Wall M.J., Wigmore G., Lopatár J., Frenguelli B.G., Dale N. (2008). The novel NTPDase inhibitor sodium polyoxotungstate (POM-1) inhibits ATP breakdown but also blocks central synaptic transmission, an action independent of NTPDase inhibition. Neuropharmacology.

[36] Zheng J., Liu Q., Yang J., Ren Q., Cao W., Yang J., Yu Z., Yu F., Wu Y., Shi H. (2012). Co-culture of apoptotic breast cancer cells with immature dendritic cells: A novel approach for DC-based vaccination in breast cancer. Braz. J. Med. Biol. Res..

[37] Kawano M., Tanaka K., Itonaga I., Iwasaki T., Miyazaki M., Ikeda S., Tsumura H. (2016). Dendritic cells combined with doxorubicin induces immunogenic cell death and exhibits antitumor effects for osteosarcoma. Oncol. Lett..

[38] Yang R., Elsaadi S., Misund K., Abdollahi P., Vandsemb E.N., Moen S.H., Kusnierczyk A., Slupphaug G., Standal T., Waage A. (2020). Conversion of ATP to adenosine by CD39 and CD73 in multiple myeloma can be successfully targeted together with adenosine receptor A2A blockade. J. Immunother. Cancer..

[39] Sharma P., Hu-Lieskovan S., Wargo J.A., Ribas A. (2017). Primary, Adaptive, and Acquired Resistance to Cancer Immunotherapy. Cell.

[40] Zhang B., Cheng B., Li F.S., Ding J.H., Feng Y.Y., Zhuo G.Z., Wei H.F., Zhao K. (2015). High expression of CD39/ENTPD1 in malignant epithelial cells of human rectal adenocarcinoma. Tumour. Biol..

[41] Künzli B.M., Berberat P.O., Giese T., Csizmadia E., Kaczmarek E., Baker C., Halaceli I., Büchler M.W., Friess H., Robson S.C. (2007). Upregulation of CD39/NTPDases and P2 receptors in human pancreatic disease. Am. J. Physiol. Gastrointest. Liver Physiol..

[42] Michaud M., Martins I., Sukkurwala A.Q., Adjemian S., Ma Y., Pellegatti P., Shen S., Keep O., Scoazec M., Mignot G. (2011). Autophagy-dependent anticancer immune responses induced by chemotherapeutic agents in mice. Science.

[43] Moesta A.K., Li X.Y., Smyth M.J. (2020). Targeting CD39 in cancer. Nat. Rev. Immunol..

[44] Kepp O., Loos F., Liu P., Kroemer G. (2017). Extracellular nucleosides and nucleotides as immunomodulators. Immunol. Rev..

[45] Castillo S.D., Sanchez-Cespedes M. (2012). The SOX family of genes in cancer development: Biological relevance and opportunities for therapy. Expert Opin. Ther. Targets.

[46] Perrot I., Michaud H.A., Giraudon-Paoli M., Augier S., Docquier A., Gros L., Courtois R., Déjou C., Jecko D., Becquart O. (2019). Blocking Antibodies Targeting the CD39/CD73 Immunosuppressive Pathway Unleash Immune Responses in Combination Cancer Therapies. Cell Rep..

[47] Li X.Y., Moesta A.K., Xiao C., Nakamura K., Casey M., Zhang H., Madore J., Lepletier A., Aguilera A.R., Sundarrajan A. (2019). Targeting CD39 in Cancer Reveals an Extracellular ATP- and Inflammasome-Driven Tumor Immunity. Cancer Discov..

