# Supplementary material for: Glioma Stem Cells Upregulate CD39 Expression to Escape Immune Response through SOX2 Modulation
Source: Cancers (Basel). 2022 Feb 3;14(3):783. doi: 10.3390/cancers14030783 (PMC8834269; doi:10.3390/cancers14030783)
Supplement: Supplementary file 1 [file cancers-14-00783-s001.zip › cancers-1581253-supplementary.pdf]

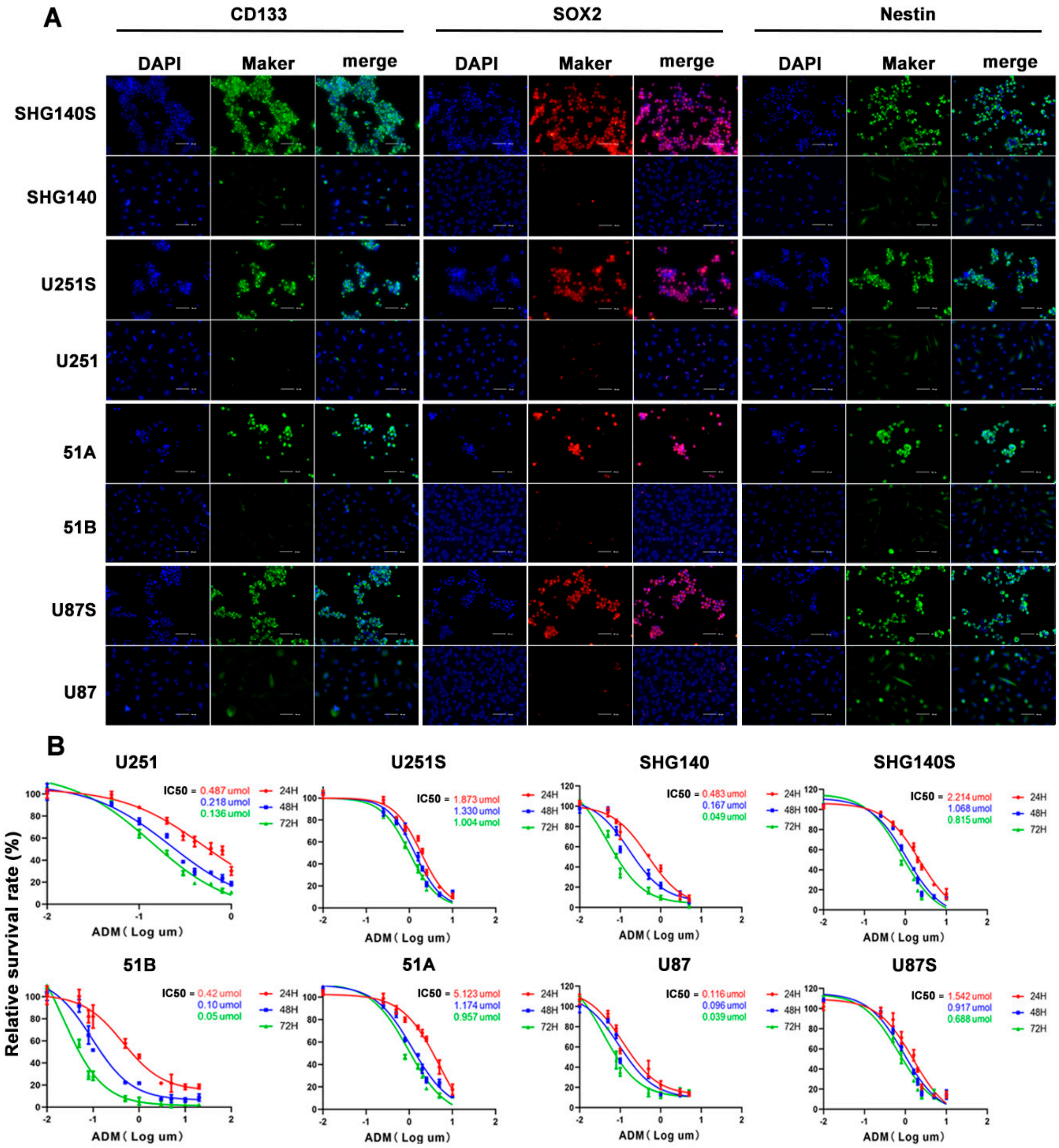

Figure S1. GSCs presented more stem cell markers and were more resistant for chemotherapy than non-GSCs. (**A**) Stem cell markers of GSCs and matched non-GSCs were detected by immunofluorescence. Scale bar = 40 μm. (**B**) GSCs and matched non-GSCs were treated with ADM, and IC50 was calculated. Data are represented as the mean ± s.d., *n* = 3. Concentration statistics were obtained with 95% confidence interval (CI). Data were analyzed by log-Nonlin fit.

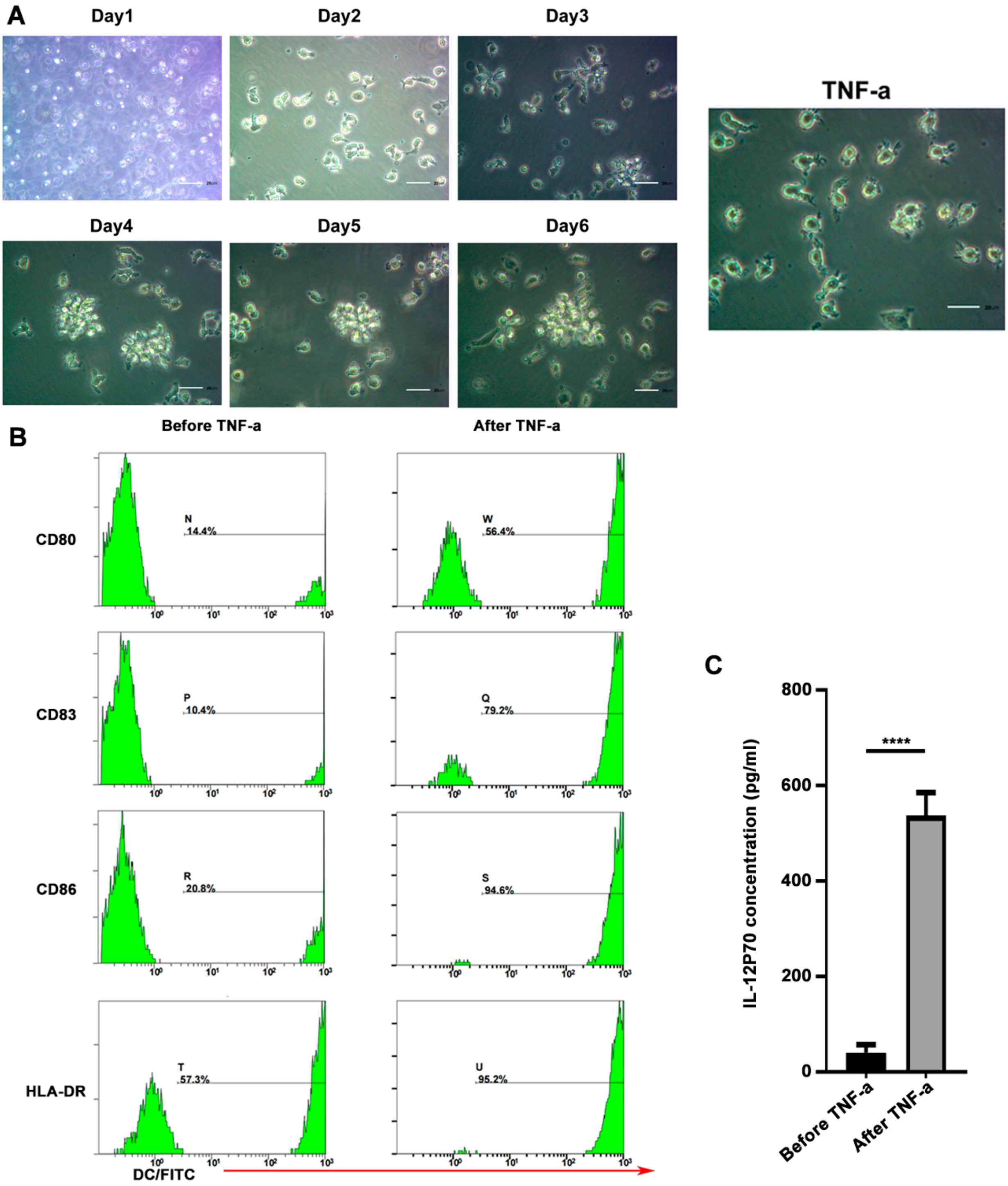

Figure S2. PBMCs were induced into functionally mature DCs after 1 week. (**A**) The morphological transformation of DCs was observed under a microscope on days 1–7. Scale bar corresponds to 20 µm. (**B**) After 24 h of intervention with TNF-a, the functional transformation of DCs was analyzed by flow cytometry. (**C**) Cytokine production by DCs stimulated with addition of TNF-a analyzed by ELISA. Data are shown as the mean ± s.d., $n = 3$, **** $p < 0.0001$, Student's *t*-test.

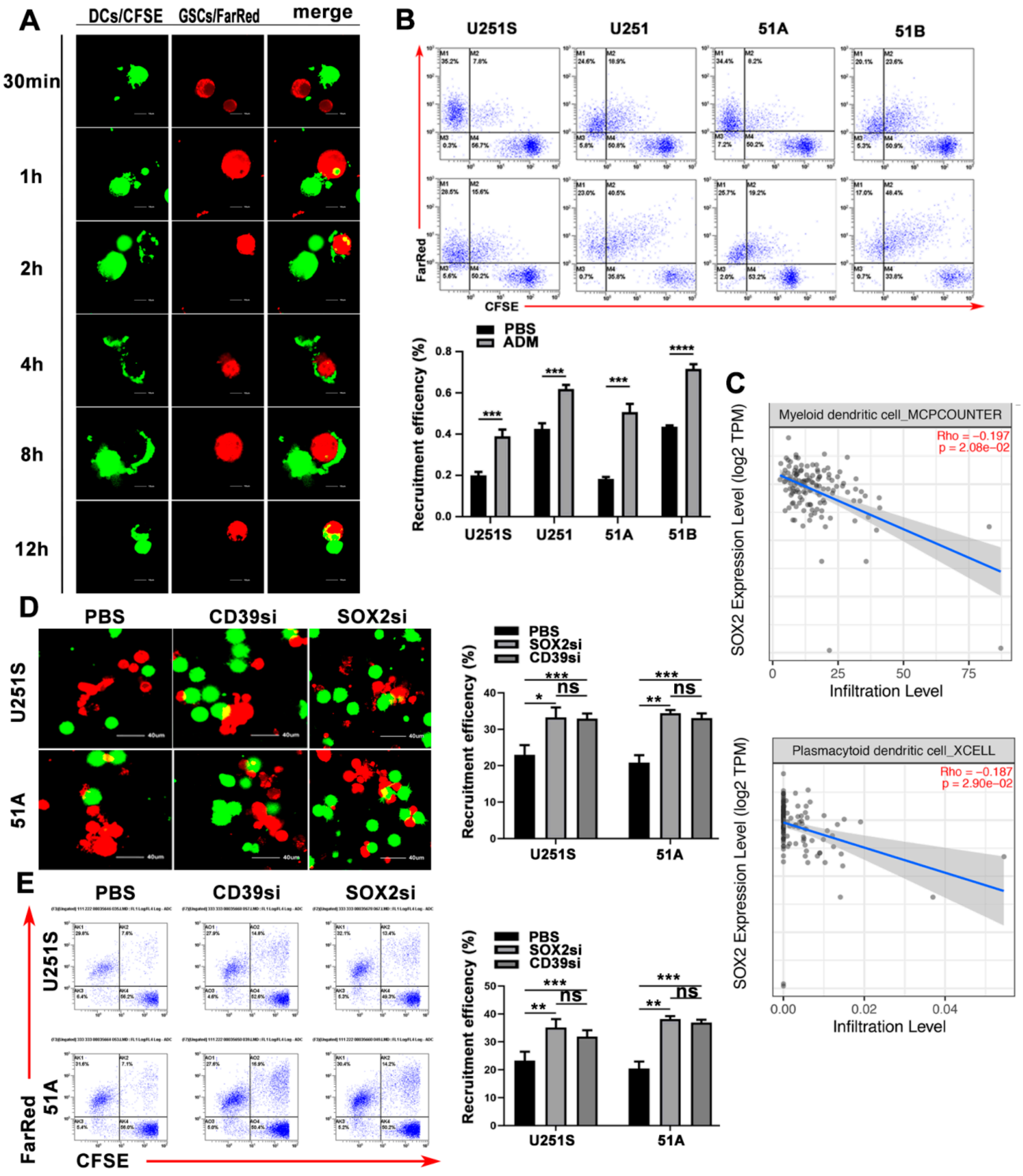

Figure S3. DCs gradually approach and phagocytize the GSCs. (**A**) The recruitment and phagocytosis of DCs were observed by confocal microscope. Scale bar corresponds to 10 μm. (**B**) Flow cytometry analysis of the ability of DCs to recruit GSCs and matched non-GSCs. (**C**) The expression of SOX2 in glioma cells and the infiltration curve of DCs were analyzed by TIMER database. DCs were co-cultured with GSCs after intervention for 12 h, and the recruitment and phagocytosis of DCs were analyzed by confocal microscope (**D**) and flow cytometry (**E**). DCs were labeled by CFSE (green), and GSCs were labeled by Far Red (red). Scale bar = 40 μm, Data are shown as the mean ± s.d., n = 5, NSP > 0.05, * $p < 0.05$, ** $p < 0.01$, *** $p < 0.001$, **** $p < 0.0001$, Student's t-test.

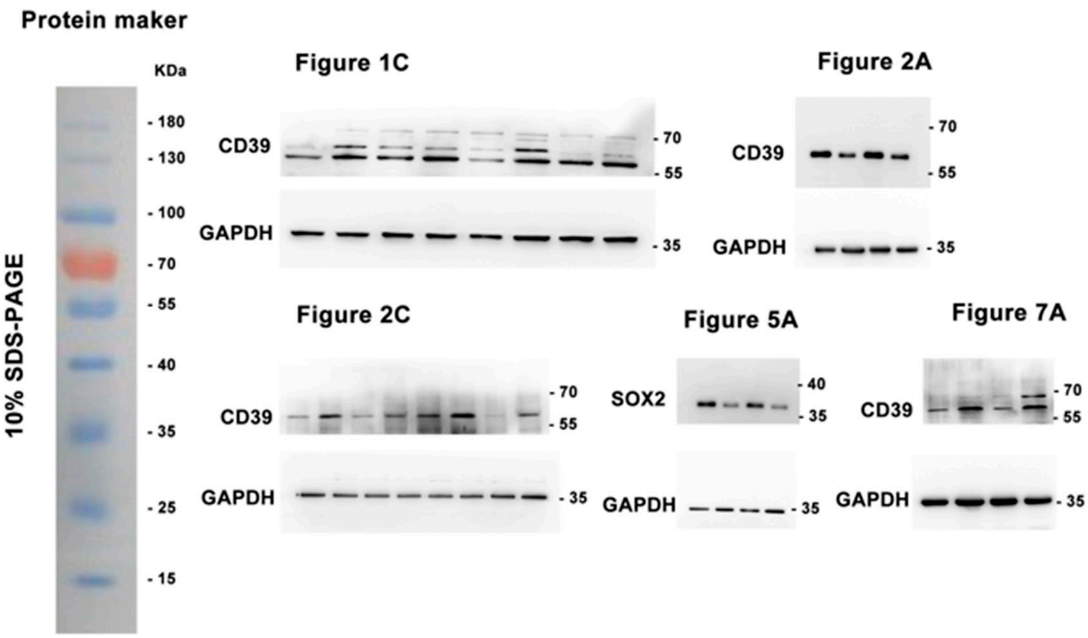

Figure S4. Uncropped Western Blots.
